# Bridging Social Circles: Need for Cognition, Prejudicial Judgments, and Personal Social Network Characteristics

**DOI:** 10.3389/fpsyg.2017.01251

**Published:** 2017-07-25

**Authors:** Petru L. Curşeu, Jeroen P. de Jong

**Affiliations:** ^1^Department of Psychology, Babeş-Bolyai University Cluj-Napoca, Romania; ^2^Department of Organisation, Open University of the Netherlands Heerlen, Netherlands

**Keywords:** need for cognition, social networks, prejudicial judgments, social attitudes

## Abstract

Various factors pertaining to the social context (availability of plausible social contacts) as well as personality traits influence the emergence of social ties that ultimately compose one’s personal social network. We build on a situational selection model to argue that personality traits influence the cognitive processing of social cues that in turn influences the preference for particular social ties. More specifically, we use a cross-lagged design to test a mediation model explaining the effects of need for cognition (NFC) on egocentric network characteristics. We used the data available in the LISS panel, in which a probabilistic sample of Dutch participants were asked to fill in surveys annually. We tested our model on data collected in three successive years and our results show that people scoring high in NFC tend to revolve in information-rich egocentric networks, characterized by high demographic diversity, high interpersonal dissimilarity, and high average education. The results also show that the effect of NFC on social network characteristics is mediated by non-prejudicial judgments.

“No man is an island, entire of itself”

(Jon Donne, 1624)

## Introduction

We live our lives in intricate social circles. The social networks in which each person is embedded shape their upbringing (Morrow, 1999), are sources of social support and buffers against mental illness at older age (Fiori et al., 2006). Throughout life, the structure of one’s social network is related to marital satisfaction and divorce (McDermott et al., 2013), career success (Seibert et al., 2001) and substance abuse (Hawkins et al., 1992). Our personal social ties are therefore inescapable forces with pervasive influences across life domains and stages and it is important to understand the emergence of one’s social ties (or in more technical terms the egocentric network structure).

The interest in exploring the emergence of social networks increased significantly during the last decades, yet research on the role of individual differences as antecedents of social network structure is rather scant (Kalish and Robins, 2006; Fiske, 2012). In their theorizing about the link between personality and social structure, Jackson and Poulsen () argue that mechanisms of social selection and social evocation explain how stable individual differences influence the egocentric network structure. The situational selection mechanism means that individual differences drive people to select their social contacts and build their social ties. In other words people select social ties that fit their personality structure. The situational evocation mechanism suggests that people actually change their social environment through their very presence. Both mechanisms point therefore toward a close association between stable individual differences and one’s position in social networks. Ryff (1987) rightfully argued that the stream of research exploring this association was not mainstream in social psychological research in the 80s and a more recent overview of research to date (Cortina et al., 2012) supports this view reporting rather limited attempts to explore the way in which individual differences shape the emergence and maintenance of social ties.

Need for cognition (NFC) is a personality trait indicative of cognitive motivation and reflects one’s inclination to extensively seek and process information (Cacioppo et al., 1996; Sohlberg, 2015). As social ties are often the vehicles through which individuals acquire and disseminate information (Haythornthwaite, 1996; Lee, 2014), the composition of one’s social network is shaped to serve his/her information needs. In light of these arguments, people scoring high in NFC will ultimately prefer information-rich social networks. We argue that such information-rich social structures are *demographically diverse* (diversity generates a rich pool of knowledge and expertise), *include distal social ties* (others different than the self are often sources of novel information) and *highly educated individuals* (educated individuals are better informed).

The emergence of social ties is also influenced by stereotypes and prejudice (Levy, 1999) and as such the composition of egocentric social network is likely to be influenced by prejudicial judgments. We extend the argument that individual differences predispose individuals to structure their social environment (Jackson and Poulsen, 2005; Kalish and Robins, 2006) and we posit that prejudicial judgments mediate the influence of cognitive motivation (NFC) on social network composition. As immigration significantly increases and Western European countries are increasingly diverse (Scuzzarello, 2012), it is important to understand the factors that influence social integration in communities and in particular the role played by individual differences (Gracia and Herrero, 2004). Therefore, we aim to extend research exploring the association between stable individual differences and social structure and this paper represents a first integrative attempt to test the extent to which the impact of NFC on social network’s characteristics is mediated by prejudicial social judgments (evaluative cognitions, rooted in negative stereotypes toward out-group members).

### Theory and Hypotheses

People navigate in their social world in different ways, and stable individual differences are likely to play an important role in the type of social relations one seeks and maintains (Jackson and Poulsen, 2005; Kalish and Robins, 2006). The situational selection mechanisms (Jackson and Poulsen, 2005) stipulates that cognitive processes translate stable individual differences in behavioral tendencies manifested in the social environment that ultimately shape the generic structure of one’s social ties. We build on the literature connecting personality with social cognition to argue that cognitive motivation (NFC) influences the processing of social information and in turn impacts one’s pattern of social relations.

Egocentric networks describe the multitude of social ties a particular actor establishes and maintains and are the vehicles through which individuals acquire and disseminate information and other socially valued resources (Haythornthwaite, 1996). In other words, egocentric networks represent one’s (social) information environment with a structure molded on individual needs and preferences. The Elaboration-Likelihood Model (ELM) of persuasion (Petty and Cacioppo, 1986; Petty et al., 1994) introduces NFC as a motivational attribute that drives in-depth and extensive processing of incoming messages. The ELM distinguishes between the central processing route (the content of the messages is well-scrutinized and understood) and peripheral processing route (the content of the messages is superficially processed and heuristics are used to oversimplify the message). Individuals with high cognitive motivation (NFC) are more inclined to use the central rather than the peripheral processing route. More recent research distinguishes between explicit and implicit NFC (Fleischhauer et al., 2013; Strobel et al., 2015), with explicit NFC influencing bottom-up target processing. Because explicit NFC is associated with more effective preconscious information processing, it may also influence the accuracy of social perception processes and will ultimately impact on preferred social ties. We therefore argue that NFC is a motivational trait that predicts the predisposition to establish information-rich egocentric networks.

### NFC and Information-Rich Egocentric Networks

As argued before, people scoring high on NFC are motivated to establish social ties that maximize their access to information/knowledge (Verplanken et al., 1992; Verplanken, 1993; Curşeu, 2011). At least three compositional features of egocentric social networks are conducive for offering access to rich cognitive resources: level of education, demographic diversity and interpersonal similarity.

Education is one of the core components of human capital (Robeyns, 2006). Educated individuals are more knowledgeable and better equipped to use and transfer their knowledge, skills and expertise across different life domains, from the job to the health and income domains (National Research Council, 2013). Through education people acquire important knowledge and expertise, therefore having ties with highly educated individuals expands one’s knowledge repertoire and access to relevant information (Curşeu et al., 2012). We build on the education as competence and human capital frameworks (Robeyns, 2006) to argue that social networks composed of highly educated members are an important knowledge repositories and people scoring high in NFC seek to establish and maintain ties with highly educated individuals.

Moreover, the “value in diversity hypothesis” (Swann et al., 2004, p. 9) states that groups or social networks composed of individuals with diverse attributes generate a substantial pool of diversified knowledge and members of diverse networks may tap into this diversified and rich knowledge repository. Therefore, direct ties with demographically diverse network members maximizes the informational input stemming from differences in life experiences among these members. As each network member revolves in other social networks, demographically diverse egocentric networks are likely to have a wide external network range, and provide access to substantial cognitive resources through these indirect ties (Reagans and McEvily, 2003; Curşeu et al., 2012). Finally, having ties with dissimilar individuals further expands one’s external network range and the access to values cognitive resources. Establishing ties with dissimilar others is illustrated by the interpersonal dissimilarity in one’s egocentric network.

To summarize, interpersonal ties are an important source of information in day-to-day life and because people scoring high in NFC seek complexity, enjoy cognitive endeavors and are open to new experiences we expect that they tend to revolve in information-rich social networks characterized by high demographic diversity, high interpersonal dissimilarity and high average education. We therefore hypothesize that:

Hypothesis 1: *NFC has a positive impact on (1) ego’s social network diversity, (2) the average interpersonal dissimilarity in ego’s social network and (3) the average education in ego’s social network.*

### The Mediating Role of Prejudicial Judgments

A core area of research on NFC concerns its influence on attitude development and change. According to the ELM (Petty and Cacioppo, 1986) people who are motivated and able to engage in laborious information processing activities, are more likely to develop accurate and unbiased representations of the (social) world (central processing route). People who lack cognitive motivation engage in heuristic information processing and are more susceptible to judgmental biases (peripheral processing route). Recent literature extended the role of cognitive motivation (NFC) from persuasion to social cognition and shows that low NFC is an important precursor of social attitudes like acceptance of stereotyping (Carter et al., 2006) and racism (Tam et al., 2008). In social-cognitive terms, stereotypes are peripheral cues that influence social perception (Levy, 1999; Carter et al., 2006). Stereotyping oversimplifies social information related to out-groups and favors (negative) prejudicial judgments toward the members of the out-groups. Crawford and Skowronski (1998) argue that exposure to social stereotypes influence the cognitive processes of people scoring high in NFC in different ways than the ones of those scoring low in NFC. Although, motivated social perceivers (people scoring high in NFC) have an enhanced recollection of stereotype-consistent information their social judgments seem to be less prejudicial when compared to those of non-motivated social-perceivers (people scoring low in NFC) (Crawford and Skowronski, 1998).

These differences (associated with cognitive motivation) in the cognitive processing of social stereotypes raise the challenge of exploring in real social settings the extent to which prejudicial attitudes mediate the impact of NFC on actual social behavior. In line with Levy (1999) we argue that people scoring high in NFC are mature social perceivers, that process incoming social information in complex ways and as such they show higher levels of interpersonal (diversity) acceptance when establishing their social networks. As a consequence of their systematic way of processing social information and their lower prejudice, people scoring high in NFC are likely to reach out to diverse others and as such revolve in more diverse social networks as compared to people scoring low in NFC.

Previous research has also shown that individuals scoring high in NFC are better able to use different others as sources of unique information in their information search efforts than those low in NFC (Kearney et al., 2009; Curşeu, 2011). Building on the fact that NFC positively correlates with openness to experience (Fleischhauer et al., 2010), fosters external information search effort (Verplanken et al., 1992; Verplanken, 1993) and decreases prejudicial behavior (Levy, 1999; Tam et al., 2008) we expect that people scoring high in NFC, due to their non-prejudicial nature actively seek for dissimilar others as information sources. We therefore hypothesize that:

Hypothesis 2: *Non-prejudicial judgments mediate the impact of NFC on (1) ego’s social network diversity, (2) the average interpersonal dissimilarity in ego’s social network and (3) the average education in ego’s social network.*

## Materials and Methods

### Ethics Statement

For the present study, no new data were collected among human participants and all data used in this study were obtained from publicly available data-bases, therefore there was no need to obtain ethical clearance from the institutional IRB.

### Participants

The paper used data from the Longitudinal Internet Studies for the Social Sciences (LISS) panel, part of CentERdata, a comprehensive survey conducted in The Netherlands. The participants were selected based on a true probability sample of households in The Netherlands and filled out the questionnaires online. In a cross-lagged design, we used three waves of the LISS panel data, we used the anonymous individual code to match the answers across the three time waves. Data for the independent and control variables were collected in a wave conducted in May 2009 and repeated in June 2009 for the non-respondents (T1, target population 8078, response rate 69.9%). The mediator variable is obtained from a “Politics and values” survey conducted in December 2009 and repeated for the non-respondents in January and February 2010 (T2, target population 9398, response rate 68%). Finally, the dependent variables were obtained from a “Social integration and leisure” survey conducted in February 2011 and repeated in March 2011 for non-respondents (T3, target population 8844, response rate 63%). The total sample contained 8702 respondents (4445 women), with an average age of 47.16 years old (all participants were older than 18). In the analyses we have used a list wise deletion of missing data and the samples for analyses varied between 3073 and 3527 depending on the variables included in the regressions.

### Measures

#### Independent Variable

The short variant of the NFC scale (translated into Dutch) was used at T1 to evaluate participants’ engagement and enjoyment of cognitive stimulation. Cronbach’s alpha for this scale was 0.88 and examples of items include: “I would prefer complex to simple problems” or “The notion of thinking abstractly is appealing to me” (1 = strongly disagree to 7 = strongly agree).

#### Mediator

Non-prejudicial judgments were evaluated at T2 with a composite measure consisting of 10 items that evaluated prejudicial judgments toward immigrants with six items (examples include: “It is good if society consists of people from different cultures,” “It should be made easier to obtain asylum in the Netherlands” rated from 1 = fully disagree to 5 = fully agree) and gender-related prejudicial judgments evaluated with four items (examples include: “A woman is more suited to rearing young children than a man,” “It is actually less important for a girl than for a boy to get a good education” rated from 1 = fully disagree to 5 = fully agree, items were reversed coded). Cronbach’s alpha for this scale is 0.77 and an exploratory factor analysis with a principal axis factoring revealed a principal factor explaining 31.89% of variance and all items loaded significantly on this principal factor. A high (composite) score is therefore indicative of less prevailing prejudicial judgments.

#### Dependent Variables

A name generator procedure was used to get information on close personal networks and participants received the following instruction: “We would like to focus on your closest social contacts now, to form a picture of the social relationships that people have. It is easier to answer these types of questions by considering concrete persons. For that reason, we ask that you list a number of persons close to you. If you wish, you can enter nicknames or initials, as long as you can remember who they refer to. Most people discuss important things with other people. If you look back on the last 6 months, with whom did you discuss important things? Please enter their first names below (to a maximum of 5).” Respondents were asked to report the gender, nationality and educational level for each of the mentioned social contacts. The social network’s demographic variety was computed using the Teachman’s (1980) index, for gender and nationality. Teachman’s (1980) index is commonly used in group diversity literature to evaluate the variety of group composition. The formula is: 
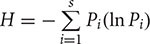
, where *i* is a particular category, *s* is the total number of categories and P_i_ is the proportion of the group members belonging to category *i*. A high value on Teachman’s index is indicative of a large gender and nationality variety within a circumscribed social group (in this case defined by the social contacts mentioned by the respondents). The two indices for gender and nationality variety were summed to obtain a general demographic diversity index of person’s social network. A high summed diversity score, reflects a diverse personal social network in term of gender and nationality.

Moreover, the participants were asked to estimate the degree of familiarity (interpersonal dissimilarity) among the nominated social contacts. A paired comparison for the five names was used to evaluate within network interpersonal dissimilarity. The answers to the 10 items of the paired comparison (person 1 compared to 2, 1 to 3, etc.) were recorded as follows: 1 = very close, 2 = not close but not stranger to each other either, and 3 = total strangers to each other). An index of interpersonal dissimilarity was computed by summing the answers to the 10 items, a high score reflects a large interpersonal distance in one’s social network. Cronbach’s alpha for this scale is 0.84 and a low score indicates that in general the members of the egocentric social network are very familiar to each other. The average education of the nominated persons was used as indicator of education in one’s egocentric social network and a high score indicates that the person seeks advice from well educated individuals (for the coding of education categories see the section on Control Variables). Social network variables were all measured at T3.

#### Control Variables

Several demographic variables were used as controls: gender (1 = male, 2 = female), age, level of education (the categories described in the Dutch Central Bureau of Statistics were used 1 = primary school, 2 = intermediate secondary education, 3 = higher secondary education, 4 = intermediate vocational education, 5 = higher vocational education, 6 = university education), place of residence (1 = extremely urban to 5 = not urban). Moreover, to control for social desirability we used the short form (MC2 – 10 items) of the Marlowe Crowne Scale (Strahan and Gerbasi, 1972). Cronbach’s alpha for this scale is 0.53 and examples of items include “I am always courteous, even to people who are disagreeable” and “I have never intensely disliked anyone” (1 = no, 2 = yes). Because the dependent variables in the study are social network variables, we also controlled for the tendency to connect to others and used Inclusion of Others in the Self Scale (IOS, Aron et al., 1992) a single-item pictorial measure of closes between self and others. All control variables were evaluated at Time 1.

## Results

Means, standard deviations, and correlations are presented in **Table 1**.

**Table 1:** Means, standard deviations, and correlations among the model variables.

	Mean	*SD*	1	2	3	4	5	6	7	8	9	10
(1) Gender (T1)	1.51	0.50	1									
(2) Age (T1)	47.16	16.87	-0.02	1								
(3) Area of residence (T1)	2.98	1.28	-0.02	0.04**	1							
(4) Education (T1)	3.75	1.62	-0.06**	-0.07**	-0.10**	1						
(5) MCS (T1)	1.58	0.199	0.04**	0.27**	0.05**	-0.07**	1					
(6) IOS (T1)	4.81	1.47	0.16**	0.14**	0.04**	-0.07**	0.19**	1				
(7) NFC (T1)	4.32	0.91	-0.16**	-0.07**	-0.10**	0.31**	-0.02	-0.01	1			
(8) NPSJ (T2)	3.31	0.51	0.17**	-0.05**	-0.11**	0.23**	0.00	0.08**	0.24^∗∗^	1		
(9) Demographic variety of SN (T3)	0.49	0.37	0.04**	-0.09**	-0.05**	0.07**	-0.05**	0.01	0.06^∗∗^	0.12^∗∗^	1	
(10) Interpersonal dissimilarity in SN (T3)	10.77	7.38	0.09**	-0.11**	-0.04**	0.11**	-0.08**	0.02	0.10^∗∗^	0.15^∗∗^	0.16^∗∗^	1
(11) Average education in SN (T3)	4.65	1.18	0.00	-0.06**	-0.13**	0.36**	-0.08**	-0.05**	0.24^∗∗^	0.26^∗∗^	0.07^∗∗^	0.10^∗∗^

*Pearson correlation coefficients are presented in the table; NFC, need for cognition; MCS, Marlowe Crowne Scale; NPSJ, non-prejudicial social judgments; SN, social network; IOS, inclusion of others in the self; Gender was coded as 1 = male, 2 = female; T1 = time 1, T2 = time 2, T3 = time 3; ^∗^*p* < 0.05; ^∗∗^*p* < 0.01; ^∗∗∗^*p* < 0.001*.

As shown in **Table 1**, NFC is positively and significantly correlated with all three social network characteristics and non-prejudicial judgments are also positively associated with the demographic diversity, interpersonal dissimilarity and average education of the egocentric networks. Moreover, the social desirability scale (MCS) negatively correlates with the three network indices and rural area residents seem to report social ties with less diverse, more similar, and less educated people than residents in urban areas. Hierarchical multiple OLS regression was conducted to test the main effect of NFC on the dependent variables. Several variables (gender, age, level of education, inclusion of others into self) expected to be associated with NFC and the dependent variables were entered in the first step (Model 1) of the regression as control variables. Area of residence was also entered as a control because it predicts stereotyping and chances of intergroup contact (in particular with foreigners). Moreover, we controlled for social desirability in order to reduce the chances that the results are impacted by respondents’ tendency to give socially desirable answers. In the second step, we have also added the mediator variable, namely the non-prejudicial social judgments (Model 2). The results of the OLS regression analyses are presented in **Table 2**. As indicated by the positive and significant effects, NFC is positively associated with the demographic diversity (β = 0.04, *t* = 2.40, *p* = 0.02), interpersonal dissimilarity (β = 0.08, *t* = 4.50, *p* < 0.001) and average education (β = 0.15, *t* = 8.81, *p* < 0.001) of the egocentric social network. Although the effect sizes are rather small for demographic diversity and interpersonal dissimilarity, we can conclude that Hypothesis 1 was supported by the data.

**Table 2:** Results of the OLS regression analyses.

	Demographic variety of SN		Interpersonal dissimilarity in SN		Average education SN	
	Model 1	Model 2	Model 1	Model 2	Model 1	Model 2
Gender	0.05**	0.03	0.12***	0.10***	0.07***	0.03*
Age	-0.07***	-0.07***	-0.07***	-0.07***	0.02	0.01
Area of residence (rural)	-0.03ˆ†	-0.03ˆ†	-0.01	-0.00	-0.09***	-0.09***
Education	0.06**	0.04*	0.07***	0.05***	0.32***	0.29***
MCS	-0.04*	-0.04*	-0.07***	-0.07***	-0.05**	-0.05**
IOS	0.03	0.03	0.04*	0.03	-0.02	-0.03
NFC	0.04*	0.03	0.09***	0.07***	0.15***	0.12***
NPSJ		0.09***		0.08***		0.16***
*R*^2^	0.02	0.03	0.04	0.05	0.17	0.19
*F* change	9.63***	25.40***	18.08***	17.82***	100^∗∗∗^	92.31***
*N*		3527		3073		3436

*Legend: standardized regression coefficients are presented in the table; NFC, need for cognition; MCS, Marlowe Crowne Scale; NPSJ, non-prejudicial social judgments; IOS, inclusion of others in the self; Gender was coded as 1 = male, 2 = female; Model 1 includes the main effect of NFC and the control variables, while Model 2 includes the mediator as well (NPSJ); ^†^*p* < 0.10; ^∗^*p* < 0.05; ^∗∗^*p* < 0.01; ^∗∗∗^*p* < 0.001*.

With respect to the control variables, gender had a significant influence both on social network demographic diversity (β = 0.05, *t* = 2.66, *p* = 0.008), as well as on interpersonal dissimilarity (β = 0.12, *t* = 5.54, *p* < 0.001), with women having more diverse social circles and reporting higher interpersonal dissimilarity in their personal social networks. Gender is also significantly related to education, with women reporting better educated egocentric networks than men (β = 0.07, *t* = 4.08, *p* < 0.001). Age is negatively associated both with social network diversity (β = -0.07, *t* = -4.07, *p* < 0.001) as well as with interpersonal dissimilarity (β = -0.07, *t* = -3.56, *p* < 0.001), older participants reporting less diverse and closer personal social ties. Also, as expected people living in urban areas revolve in more diverse (β = -0.03, *t* = -1.95, *p* = 0.05) and educated (β = -0.08, *t* = -5.46, *p* < 0.001) social circles, while highly educated participants report having highly educated (β = 0.32, *t* = 19.41, *p* < 0.001) and demographically diverse (β = 0.06, *t* = 3.14, *p* = 0.002) egocentric networks as well as a larger average interpersonal dissimilarity (β = 0.07, *t* = 3.74, *p* < 0.001) in their social ties. Social desirability scale negatively predicts the reported demographic diversity (β = -0.04, *t* = -2.26, *p* = 0.02), the interpersonal dissimilarity (β = -0.07, *t* = -3.68, *p* < 0.001) and the average education (β = -0.05, *t* = -2.96, *p* = 0.003) in one’s social circle.

In order to test the indirect effect of NFC on the dependent variables we have used the bootstrapping procedure described by Preacher and Hayes (2008) as well as Hayes’s PROCESS procedure for SPSS (beta release 130612). The indirect effect of NFC on the SN demographic variety is positive and significant (indirect effect size = 0.007; *SE* = 0.001 *CI_low_* = 0.005; *CI_high_* = 0.01), while the direct effect is not significant (direct effect size = 0.009, *SE* = 0.007; *t* = 1.36, *p* = 0.17). Therefore, although the indirect effect sizes are small, non-prejudicial judgments fully mediate the effect of NFC on the variety of SN. Moreover, the indirect effect of NFC on interpersonal dissimilarity is positive and significant (indirect effect size = 0.13; *SE* = 0.03; *CI_low_* = 0.07; *CI_high_* = 0.21), and the direct effect is also positive and significant (direct effect size = 0.55, *SE* = 0.15; *t* = 3.57, *p* = 0.0004). Therefore, with a small indirect effect size, non-prejudicial judgments partially mediate the effect of NFC on the interpersonal dissimilarity in the SN. Finally, the indirect effect of NFC on average education in SN is positive and significant (indirect effect size = 0.04; *SE* = 0.005; *CI_low_* = 0.03; *CI_high_* = 0.05), and the direct effect is also positive and significant (direct effect size = 0.15, *SE* = 0.02; *t* = 6.85, *p* < 0.0001). Non-prejudicial judgments partially mediate the effect of NFC on average education level in SN, therefore the second hypothesis was also supported by the data.

**FIGURE 1:**
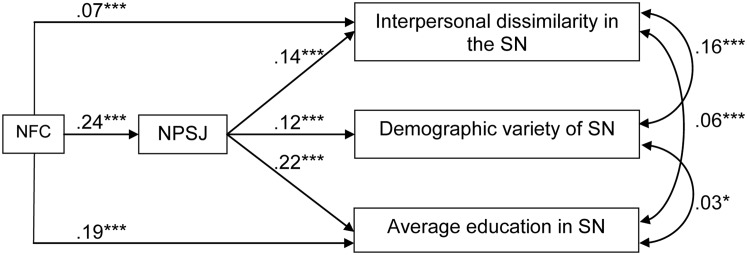
The results of the overall path model. Values represent standardized path coefficients (^∗∗∗^*p* < 0.0001, ^∗∗^*p* < 0.01, and ^∗^*p* < 0.05); NFC, need for cognition; NPSJ, non-prejudicial social judgments. Fit indices: chi-square = 5.97, df = 1, *p* = 0.01, CFI = 0.99, TLI = 0.92, NFI = 0.99, RMSEA = 0.024.

We further tested the global model and estimated the covariance between the dependent variables (that we argued reflect information-rich social network structures) using structural equation modeling in AMOS (version 21). In order to test the robustness of the mediation analysis, we did not use any of the control variables included in the bootstrapping analyses presented before. The results of the overall model fit the separate mediation analyses reported before and are presented in **Figure 1**.

The incremental fit indices reported in **Figure 1** show that the model cannot be significantly improved. Although the chi square is significant (often the case in large sample sizes) the RMSEA shows that the theoretical model fits the structure of covariances observed in the data, supporting the second hypothesis and the overall mediation claims discussed before.

## Discussion

Our study tested the extent to which prejudicial judgments mediate the effect of NFC on egocentric network characteristics. We built on the cognitive motivation literature to argue that NFC is positively associated with information search. Social networks provide important cognitive resources that people scoring high in NFC are motivated to tap into. In particular, social network diversity (demographic diversity and interpersonal dissimilarity) is a source of non-redundant knowledge in egocentric networks, while the average education is an indicator of information richness and we argued that NFC is a positive predictor of these three indicators of information-rich egocentric networks. Our results support the claim that people scoring high on NFC tend to build information rich social networks.

### Implications for Theory

The results reported in our paper extend our understanding of the interplay between personality and social structure by showing that social attitudes are one of the plausible mechanisms that explain this link (Cortina et al., 2012). Our study also shows that non-prejudicial judgments are important predictors of social network structure. First, we show that people scoring high in NFC revolve in information-rich social networks. We therefore contribute to the social selection models (Jackson and Poulsen, 2005) and show that NFC is an important precursor of egocentric network characteristics. Second, our study also supports the positive role of motivation in regulating prejudicial attitudes. These results support the observation that people scoring high on NFC are, what Levy (1999) called, “mature social perceivers” (people scoring high in NFC are motivated to better understand their social world and as such are more likely to be accepted and find a niche in social networks). They process social information in complex ways and are able to identify similarities between different social subgroups. On contrary, people scoring low in NFC are more likely to rely on stereotypes when judging incoming social information. The immediate consequence of “mature social judgments” (free of social stereotyping) is the engagement in more varied and diversified social networks. In other words, NFC is an important determinant of mature (non-prejudicial) social judgments which in turn predict the characteristics of person’s social network.

Our results show that prejudicial judgments fully mediate the impact of NFC on social network’s demographic variety. In this way, we extend the ELM (Petty and Cacioppo, 1986; Petty et al., 1994) to social cognition and show that low NFC predicts heuristic, stereotype-based social information processing. This result also extends previous literature on NFC and acceptance of stereotyping (Carter et al., 2006) by testing directly the extent to which prejudicial judgments explain the impact of NFC on social behavior. More recent empirical evidence distinguished between explicit and implicit NFC and shows that explicit NFC impacts on reflective information processing while implicit NFC impacts on impulsive information processing (Fleischhauer et al., 2013). Similarly, ample research on implicit social cognition points toward the implicit nature of stereotypes and stereotyping (Greenwald et al., 2002), therefore further research should explore the extent to which implicit stereotypes mediate the impact of explicit and implicit NFC on social behavior.

Moreover, prejudicial judgments partially mediate the impact of NFC on the interpersonal dissimilarity of social network members. NFC is a strong predictor for the interpersonal dissimilarity of social network contacts, supporting previous research, showing that people scoring high in NFC reach out for advice to out-groups and different others to a greater extent than people scoring low in NFC (Curşeu, 2011). Individuals scoring high in NFC reach out for advice to people rather unfamiliar with each other, while those scoring low in NFC seem to revolve in social networks whose members are familiar with each other. Value in diversity hypothesis (Swann et al., 2004) offers a plausible explanation for this result, namely that people scoring high in NFC recognize better than those scoring low in NFC the value of different others as informational resources.

The most important theoretical contribution of the paper is that it further extends the literature on the influence of NFC on social cognition and social interaction. The paper uses direct evaluations of prejudicial judgments adding to previous studies that explored the role of NFC on the acceptance of stereotyping (Carter et al., 2006) and shows that NFC is an important motivational factor that triggers in depth processing of social information illustrated by a lower reliance on prejudicial judgments in social perception. Ultimately, the paper shows that social cognition is the linking pin between a stable individual difference associated with engagement in cognitive endeavors and the emergence of social networks. We use data collected in a substantial sample and as most studies published in premier Social Psychology outlets use samples rarely larger than 100 participants (Fraley and Vazire, 2014) we believe that our findings are robust, although most of the direct and indirect effect sizes are small.

### Implications for Practice

The results reported here also have important practical implications for organizational team management because NFC fosters the beneficial effects of team diversity on team performance (Kearney et al., 2009) and is also associated with information search in small group settings (Curşeu, 2011). As modern organizations are increasingly diverse and most organizational groups are engaged in information sharing and information processing activities, using NFC evaluation in personnel selection, could substantially improve the team staffing practices. The two dimensions (gender and nationality) used in this study to compute the social network variety are particularly relevant as women and immigrants are occupational minorities that received considerable attention because their labor force participation in Europe steadily increased during the last decades. Mass media often depicts women and immigrants as occupational minorities and socially shared negative stereotypes may generate conflicts in the workplace that ultimately decrease group and organizational performance (Jacobs et al., 2015). It is therefore important to understand the factors that generate more social integration in social networks and ultimately in the increasingly diverse workplaces.

From a community perspective, people scoring high in NFC could act as community integrators as they seem to revolve in highly diversified social networks. Our research adds to the empirical insights on the influence of individual differences on social integration (Gracia and Herrero, 2004) and shows that NFC decreases prejudicial judgments and is the core of more integrated personal social networks. As immigrant groups tend to cluster and form geographically dense clusters in the proximity of other groups with rather similar cultural and historical background (Novotny and Hasman, 2015) their integration in localcommunities is often limited (Scuzzarello, 2012). According to our results, people scoring high in NFC seem to be social catalyzers that could facilitate the integration of migrants in local communities. Although socio-economic conditions (relative deprivation and relative wealth) are important drivers of negative attitudes toward immigrants (Jetten et al., 2015) our study points toward NFC as an important inter-individual difference that may play a moderating role in explaining the strength of the association between socio-economic status and discrimination. Further research could explore this claim.

### Limitations and Future Research Avenues

Next to its contributions, this study has several limitations as well. First, our mediator included prejudicial judgments only for two social groups and although both are highly relevant for the modern Dutch societal context (Williams et al., 1999; Curşeu et al., 2007; Velasco et al., 2008) future research could explore a broader range of prejudicial judgments. It would be for example interesting to explore whether the mediation effect works for prejudicial judgments in general (is stable across various types of prejudicial judgments) or it displays peculiarities for specific social groups. Second, the variables included in the study are self-reported and therefore the results could be influenced by common-source bias. However, because the model uses three waves of data collected over a period of 3 years and the common scale properties were removed as much as possible (Podsakoff et al., 2011) the common method bias is probably reduced, though not completely eliminated. Moreover, we have used a social desirability measure (MCS) to partial out the plausible influence of desirability in answers. Our results supported the hypotheses and reveal significant, yet most of the times small effect sizes. Future research could further explore the association between NFC and egocentric social network structure in other samples in order to get a clearer indication on the magnitude of these effect sizes. Finally, although data reported here comes from measurements separated in time, causal inferences should be interpreted with caution, as temporal precedence is a necessary and not sufficient condition for determining causal relations.

## Conclusion

Need for cognition reflects the tendency to engage in and enjoy activities involving elaborated information processing and individuals scoring high in NFC tend to revolve in information-rich social networks. Non-prejudicial attitudes are important mechanisms through which cognitive motivation (NFC) influences the emergence of egocentric network structures such that people scoring high in NFC are less prejudiced and as a consequence have more diversified and better educated egocentric networks.

## Author Contributions

Designed the study: PC, JdJ; analyzed the data: PC, JdJ; wrote the paper: PC, JdJ.

## Conflict of Interest Statement

The authors declare that the research was conducted in the absence of any commercial or financial relationships that could be construed as a potential conflict of interest.
